# Wearing Rubber Shoes

**Published:** 1859-11

**Authors:** 


### WEARING RUBBER SHOES.

THE tendency of India-rubber shoes is to make the feet cold, and in such proportion endanger health; hence, they are useful only in walking when the ground is muddy or sloshy with melting snow—in these cases they are invaluable, and there is no equal substitute. Two rules should be observed whenever it is possible: when rubbers are on the feet persons should keep moving, and remove them on entering the house, if it is intended to remain over a few minutes. If the rubbers have been on the feet several hours, both shoes and stockings are necessarily damp by the condensation and confinement of the perspiration, therefore all should be removed, and the naked foot held to the fire until warm and dry in every part; if then a pair of dry stockings are put on, and a pair of warmed and loose slippers or shoes, there will be a feeling of comfort for the remainder of the day, which will more than compensate for the trouble taken, to say nothing of the ailments averted. But it must not be forgotten that as India-rubber shoes are impervious to water from without, and ought not to be worn except in muddy weather, and only then while the wearer is in motion, so leather shoes, rendered impervious to water, by blacking or by any other means, should be used like India-rubbers, temporarily, and when walking in mud or slosh. For common purposes the old-fashioned leather boots and shoes are best, if kept well blacked, with several renewals of dry socks during the day if the feet perspire profusely. As cold and damp feet are the avenues of death to multitudes every year, a systematic attention to the above suggestions would save many a valuable life.

